# Supplementary material for: Virus-specific memory T cell responses unmasked by immune checkpoint blockade cause hepatitis
Source: Nat Commun. 2021 Mar 4;12:1439. doi: 10.1038/s41467-021-21572-y (PMC7933278; doi:10.1038/s41467-021-21572-y)
Supplement: Supplementary file 1 — Supplementary Information [file 41467_2021_21572_MOESM1_ESM.pdf]

# - SUPPLEMENTARY INFORMATION -

Virus-specific memory T cell responses unmasked by immune checkpoint blockade cause hepatitis

James A. Hutchinson, Katharina Kronenberg, Paloma Riquelme, Jürgen J. Wenzel, Gunther Glehr, Hannah-Lou Schilling, Florian Zeman, Katja Evert, Martin Schmiedel, Marion Mickler, Konstantin Drexler, Florian Bitterer, Laura Cordero, Lukas Beyer, Christian Bach, Josef Koestler, Ralph Burkhardt, Hans J. Schlitt, Dirk Hellwig, Jens M. Werner, Rainer Spang, Barbara Schmidt, Edward K. Geissler and Sebastian Haferkamp

List of Supplementary Figures

Suppl. Fig. 1 Recruitment of patients advanced melanoma into a prospective, single-center, non-randomised observational clinical trial

Suppl. Fig. 2 Gating strategy for evaluating flow cytometry data generated from human peripheral blood samples stained with Duraclone IM Phenotyping BASIC tubes

Suppl. Fig. 3 Gating strategy for evaluating flow cytometry data generated from human peripheral blood samples stained with Duraclone IM T cell Subsets tubes

Suppl. Fig. 4 Gating strategy for evaluating flow cytometry data generated from human peripheral blood samples stained with Duraclone IM T cell Receptors (TCR) tubes

Suppl. Fig. 5 Gating strategy for evaluating flow cytometry data generated from human peripheral blood samples stained with Duraclone IM Regulatory T cell (Treg) tubes

Suppl. Fig. 6 Gating strategy for evaluating flow cytometry data generated from human peripheral blood samples stained with Duraclone IM B cell tubes

Suppl. Fig. 7 Gating strategy for evaluating flow cytometry data generated from human peripheral blood samples stained with Duraclone IM Dendritic Cell (DC) tubes

Suppl. Fig. 8 Gating strategy for evaluating flow cytometry data generated from human peripheral blood samples stained with Duraclone IM Granulocytes tubes

Suppl. Fig. 9 $CD4^+$ T_{EM} cell enrichment is associated with chronic activation and expansion of effector memory $CD8^+$ T cells.

Suppl. Fig. 10 A refined prediction of hepatitis in CMV IgG^+ patients

Suppl. Fig. 11 Quantifying *in vitro*-restimulated CMV-reactive T cells by flow cytometry.

Suppl. Fig. 12 Detection of CMV-reactive TNFα- and IL-4-producing $CD4^+$ T cells in a CMV IgG^+ $CD4^+$ $T_{EM}^{\geq 16\%}$ patient who developed hepatitis.

List of Supplementary Tables

Suppl. Table 1 Baseline Biochemistry and Haematology of patients included in the training and validation sets

Suppl. Table 2 Clinical characteristics of patients included in the training and validation sets

Suppl. Table 3 Associations between common adverse reactions and clinical responses

Suppl. Table 4 Baseline Biochemistry and Haematology of patients with or without hepatitis

Suppl. Table 5 Baseline clinical characteristics of patients with or without hepatitis

Suppl. Table 6 Presence of autoimmune antibodies was not associated with development of hepatitis following αPD-1/αCTLA-4 dual therapy

Suppl. Table 7 Characteristics of patients who were classified as (i) without treatment-related hepatitis, (ii) $CD4^+$ $T_{EM}^{<21\%}$ patients who developed hepatitis, or (iii $CD4^+$ $T_{EM}^{\geq 21\%}$ patients who developed hepatitis

SUPPLEMENTARY FIGURE 1

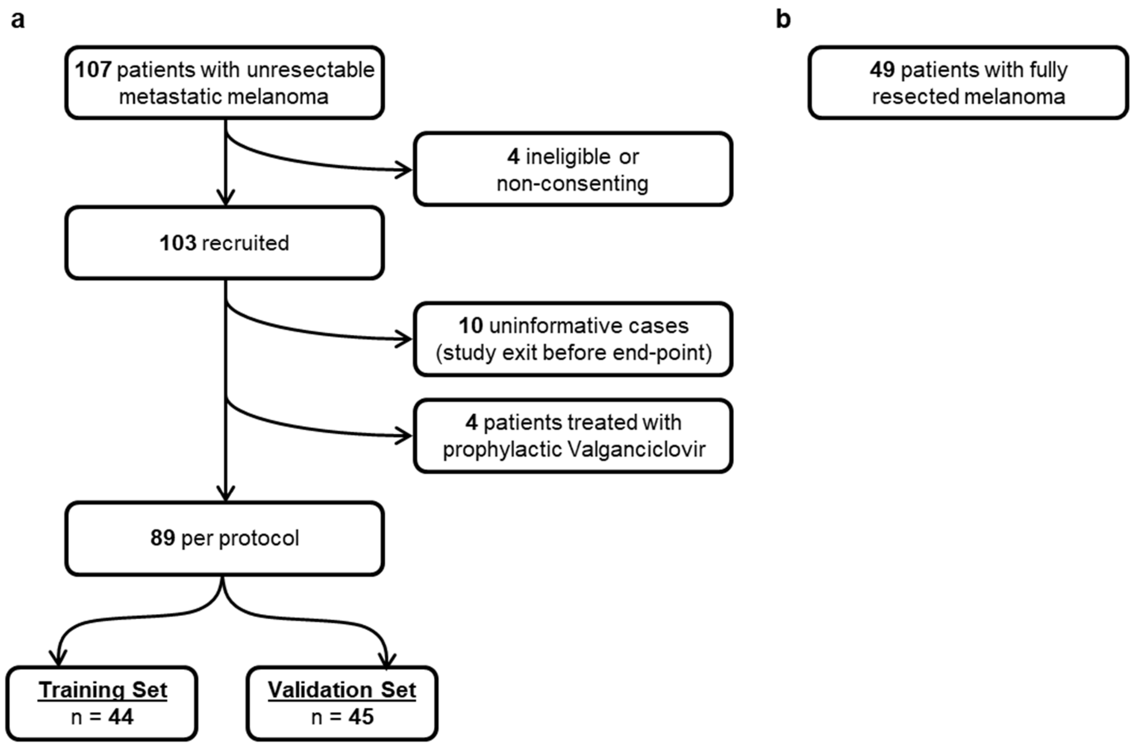

Recruitment of patients advanced melanoma into a prospective, single-center, non-randomised observational clinical trial (clinicaltrials.gov #NCT04158544). **(a)** 107 adults (>18 years) presenting with unresectable metastatic melanoma suitable for treatment with αPD-1/αCTLA-4 dual therapy were screened for trial eligibility. 103 patients were recruited in total. Of these, 10 patients were excluded from subsequent analyses because they significantly deviated from the treatment protocol or died before registering an end-point of complication-free survival to staging at 12 weeks. A further 4 patients were treated with prophylactic valganciclovir. The remaining 89 cases were randomly assigned to a training set (n=44) and validation set (n=45). **(b)** 49 adults (>18 years) with fully resected melanoma suitable for treatment with αPD-1 monotherapy therapy were enrolled as a comparator population.

SUPPLEMENTARY FIGURE 2

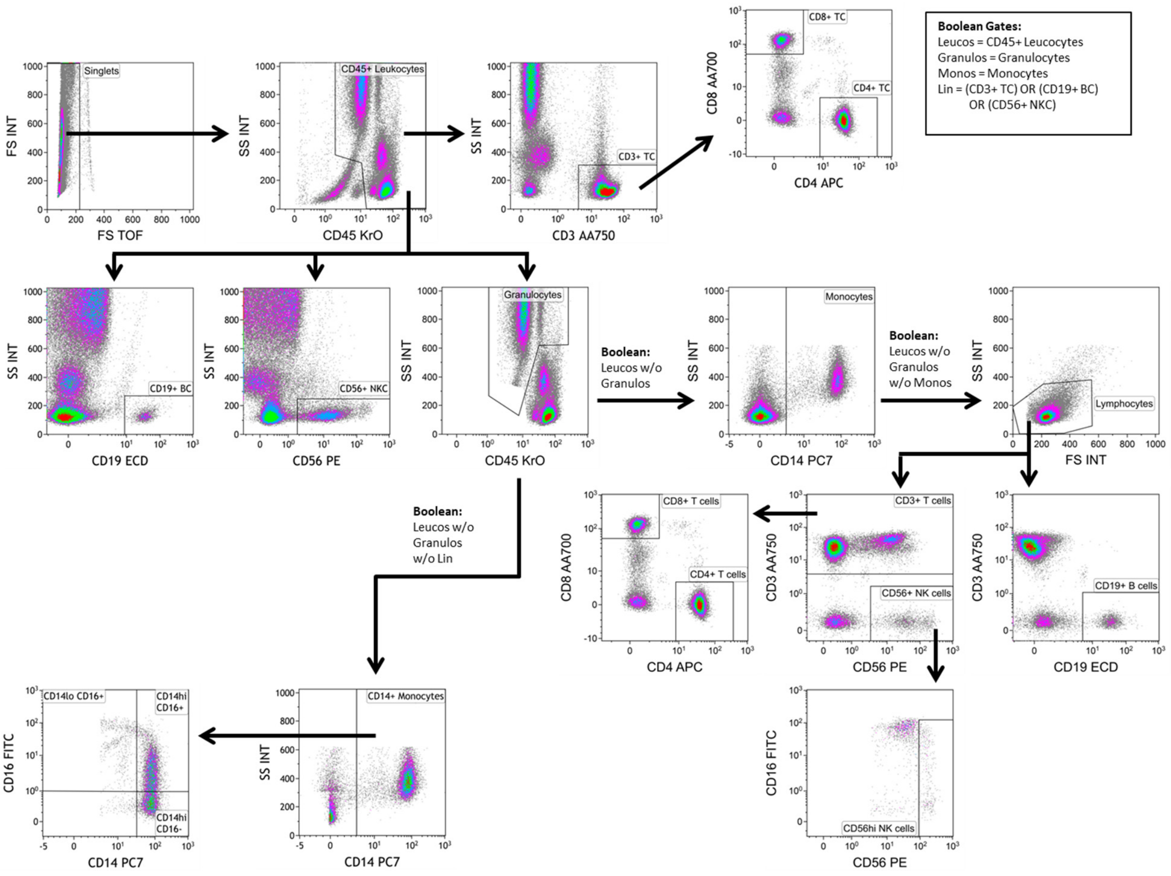

Gating strategy for evaluating flow cytometry data generated from human peripheral blood samples stained with Duraclone IM Phenotyping BASIC tubes. A detailed step-by-step procedure for preparing and analysing clinical samples by flow cytometry is available through Nature Protocol Exchange[1]. Duraclone IM Phenotyping BASIC tubes (B53309; Beckman Coulter) are pre-formulated, dried-down, 8-colour panels comprising the following monoclonal antibodies: CD16 (clone 3G8, FITC); CD56 (NG01, PE); CD19 (J3_119, ECD); CD14 (RMO52, PC7); CD4 (13B8.2, APC); CD8 (B9.11, AF700); CD3 (UCHT-1, APC-A750); CD45 (J33, Krome Orange). This gating strategy was used to generate data presented in Figures 2a & 4e.

SUPPLEMENTARY FIGURE 3

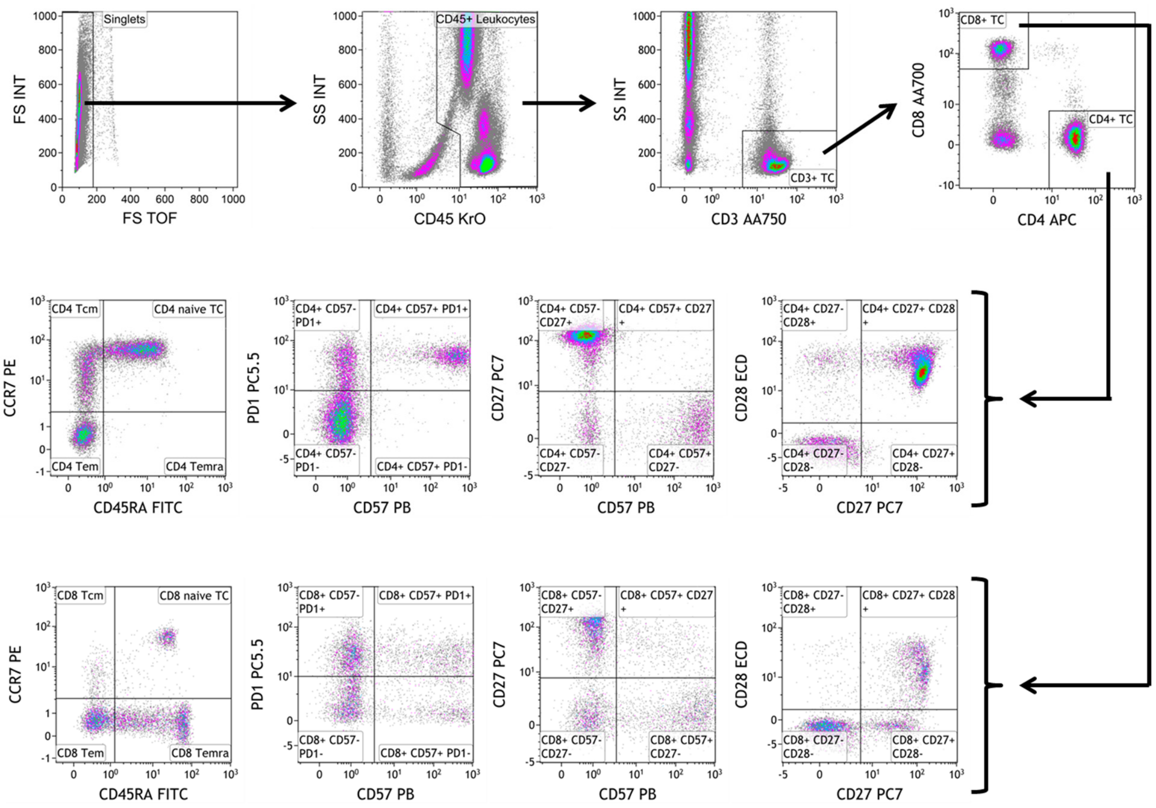

Gating strategy for evaluating flow cytometry data generated from human peripheral blood samples stained with Duraclone IM T cell Subsets tubes. A detailed step-by-step procedure for preparing and analysing clinical samples by flow cytometry is available through Nature Protocol Exchange[1]. Duraclone IM T cell Subsets tubes (B53328; Beckman Coulter) are pre-formulated, dried-down, 10-colour panels comprising the following monoclonal antibodies: CD45RA (clone 2H4, FITC); CD197 (GD43H7, PE); CD28 (CD28.2, ECD); CD279 (PD1.3.5, PC5.5); CD27 (1A4.CD27, PC7); CD4 (13B8.2, APC); CD8 (B9.11, AF700); CD3 (UCHT-1, APC-A750); CD57 (NC1, Pacific Blue); CD45 (J33, Krome Orange). This gating strategy was used to generate data presented in Figures 2a & 4e, as well as every quantification of $CD4^{+}$ T_{EM} cells in Figures 2, 3, 4, 5, 7, 8 and 9.

SUPPLEMENTARY FIGURE 4

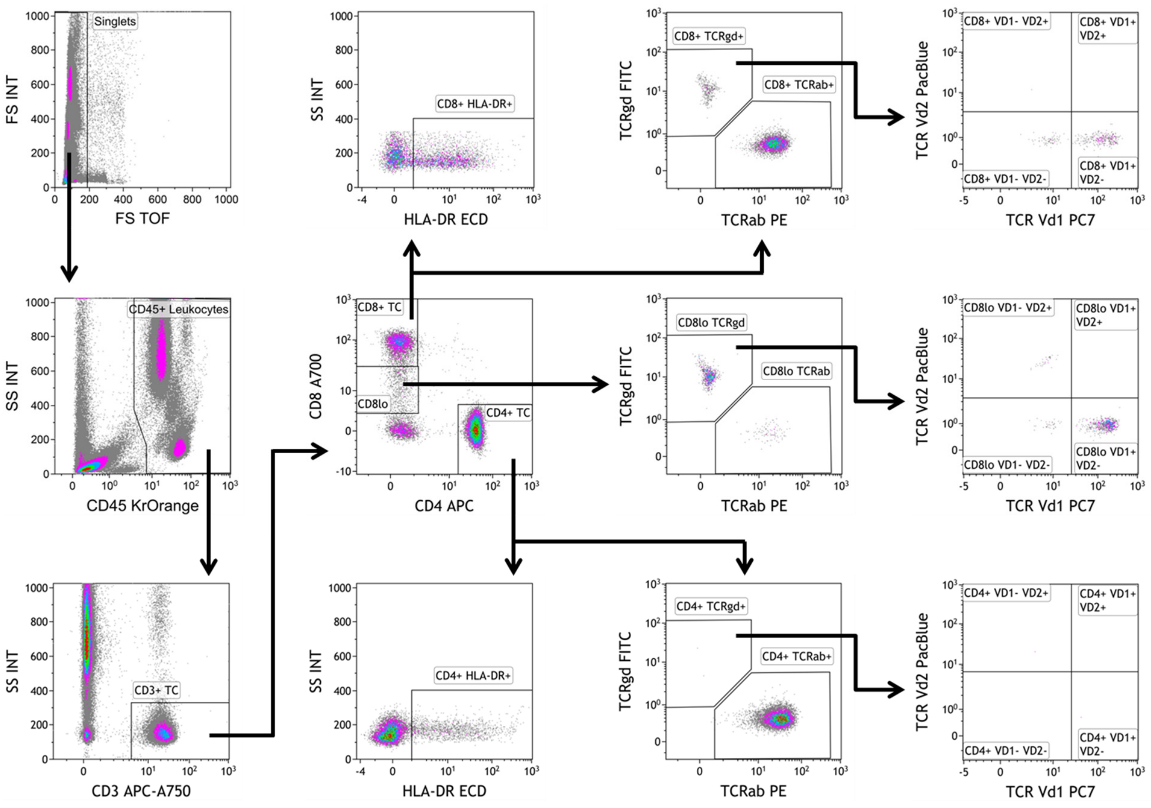

Gating strategy for evaluating flow cytometry data generated from human peripheral blood samples stained with Duraclone IM T cell Receptors (TCR) tubes. A detailed step-by-step procedure for preparing and analysing clinical samples by flow cytometry is available through Nature Protocol Exchange[1]. Duraclone IM TCR tubes (B53340; Beckman Coulter) are pre-formulated, dried-down, 9-colour panels comprising the following monoclonal antibodies: TCRγδ (IMMU510, FITC); TCRαβ (IP26A, PE); HLA-DR (Immu-357, ECD); TCR-Vδ1 (R9.12, PC7); CD4 (13B8.2, APC); CD8 (B9.11, AF700); CD3 (UCHT-1, APC-A750); TCR-Vδ2 (IMMU 389, Pacific Blue); CD45 (J33, Krome Orange). This gating strategy was used to generate data presented in Figures 2a & 4e.

SUPPLEMENTARY FIGURE 5

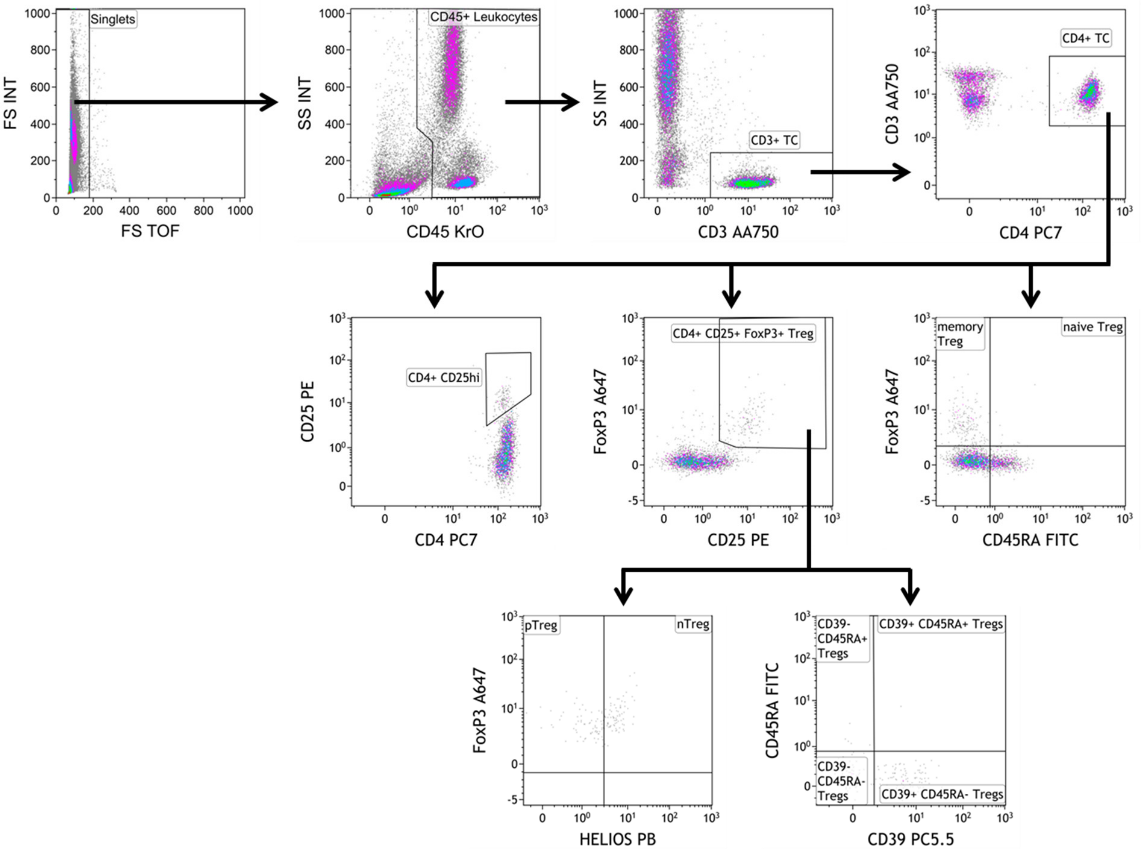

Gating strategy for evaluating flow cytometry data generated from human peripheral blood samples stained with Duraclone IM Regulatory T cell (Treg) tubes. A detailed step-by-step procedure for preparing and analysing clinical samples by flow cytometry is available through Nature Protocol Exchange[1]. Duraclone IM Treg tubes (B53346; Beckman Coulter) are pre-formulated, dried-down, 8-colour panels comprising the following monoclonal antibodies: CD45RA (clone 2H4, FITC); CD25 (B1.49.9, PE); CD39 (BA54, PC5.5); CD4 (T4, PC7); FoxP3 (259D, AF647); CD3 (UCHT-1, APC-A750); Helios (22F6, Pacific Blue); CD45 (J33, Krome Orange). This gating strategy was used to generate data presented in Figures 2a & 4e.

SUPPLEMENTARY FIGURE 6

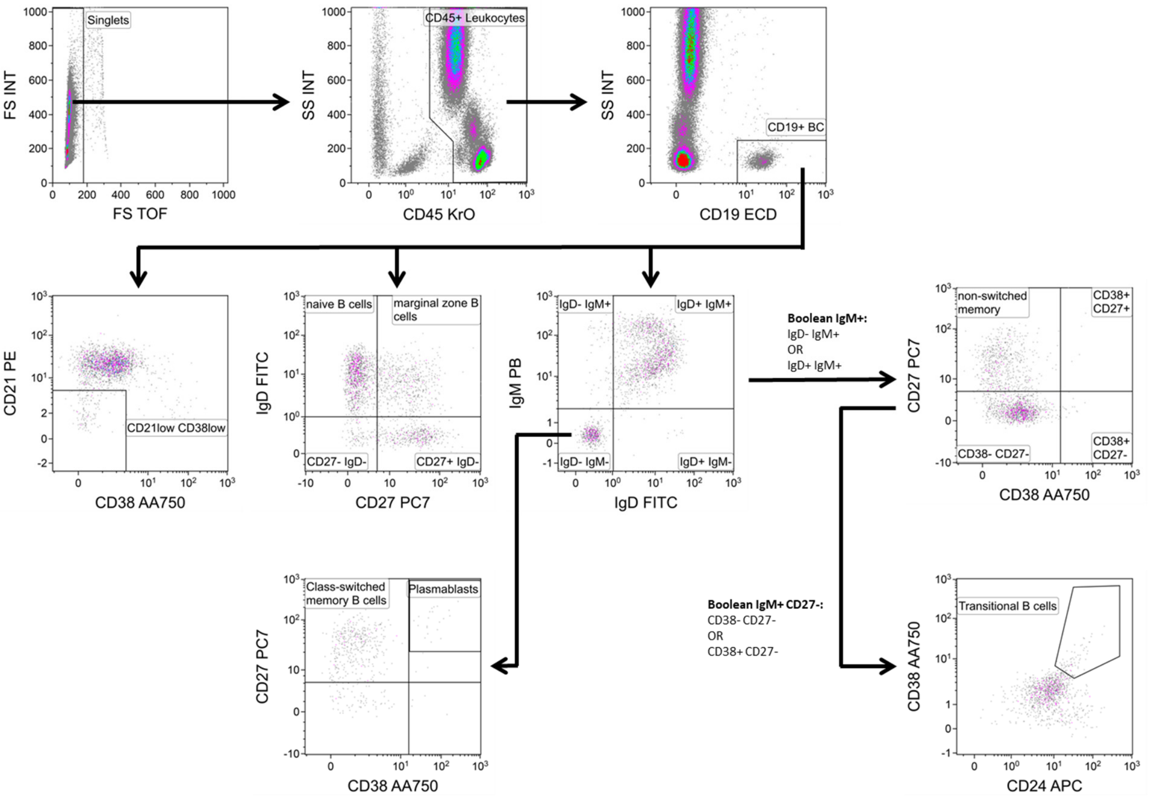

Gating strategy for evaluating flow cytometry data generated from human peripheral blood samples stained with Duraclone IM B cell tubes. A detailed step-by-step procedure for preparing and analysing clinical samples by flow cytometry is available through Nature Protocol Exchange[1]. Duraclone IM B cell tubes (B53318; Beckman Coulter) are pre-formulated, dried-down, 8-colour panels comprising the following monoclonal antibodies: IgD (1A6-2, FITC); CD21 (BL13, PE); CD19 (J3-119, ECD); CD27 (1A4CD27, PC7); CD24 (ALB9, APC); CD38 (LS198-4-3, APC-A750); IgM (SA-DA4, Pacific Blue); CD45 (J33, Krome Orange). This gating strategy was used to generate data presented in Figures 2a & 4e.

SUPPLEMENTARY FIGURE 7

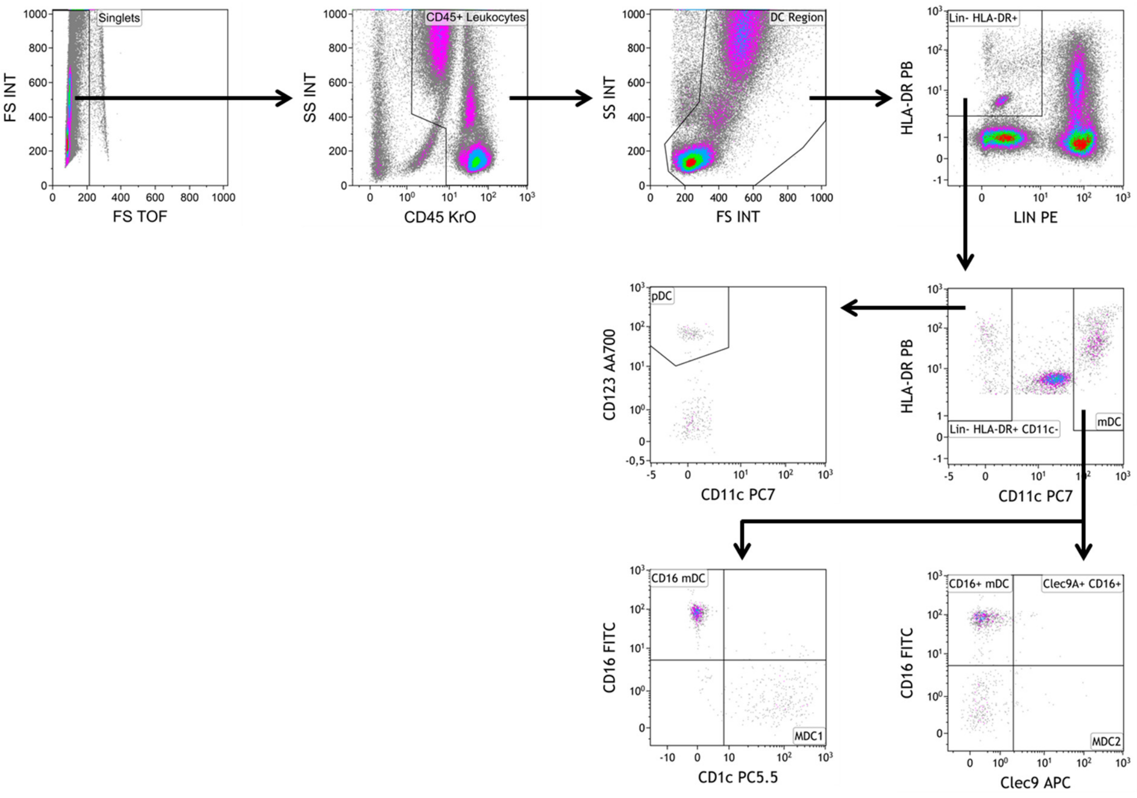

Gating strategy for evaluating flow cytometry data generated from human peripheral blood samples stained with Duraclone IM Dendritic Cell (DC) tubes. A detailed step-by-step procedure for preparing and analysing clinical samples by flow cytometry is available through Nature Protocol Exchange[1]. Duraclone IM DC tubes (B53351; Beckman Coulter) are pre-formulated, dried-down, 8-colour panels comprising the following monoclonal antibodies: CD16 (clone 3G8, FITC); CD3 (UCHT-1, PE); CD14 (RMO52, PE); CD19 (J3-119, PE); CD20 (HRC20, PE); CD56 (N901, PE); CD1c (L161, PC5.5); CD11c (BU15, PC7); Clec-9A (8F9, APC); CD123 (SSDCLY107D2, APC-A700); HLA-DR (IMMU-357, Pacific Blue); CD45 (J33, Krome Orange). This gating strategy was used to generate data presented in Figures 2a & 4e.

SUPPLEMENTARY FIGURE 8

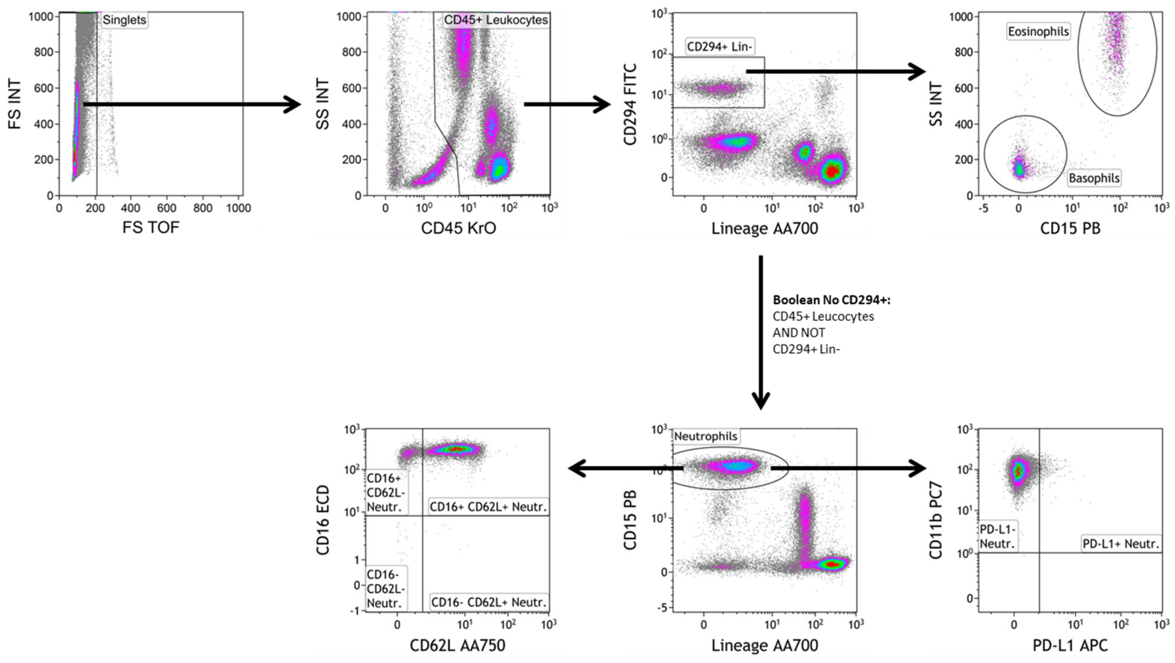

Gating strategy for evaluating flow cytometry data generated from human peripheral blood samples stained with Duraclone IM Granulocytes tubes. A detailed step-by-step procedure for preparing and analysing clinical samples by flow cytometry is available through Nature Protocol Exchange[1]. Duraclone IM Granulocytes tubes (B88651; Beckman Coulter) are pre-formulated, dried-down, 9-colour panels comprising the following monoclonal antibodies: CD294 (clone BM16, FITC); CD16 (3G8, ECD); CD33 (D3HL60.251, PC5.5); CD11b (Bear1, PC7); CD274 (PDL1.3.1, APC); CD3 (UCHT-1, APC-A700); CD19 (J3-119, APC-A700); CD56 (N901, APC-A700); CD14 (RMO52, APC-A700); CD62L (DREG56, APC-AF750); CD15 (80H5, Pacific Blue); CD45 (J33, Krome Orange). This gating strategy was used to generate data presented in Figures 2a & 4e.

SUPPLEMENTARY FIGURE 9

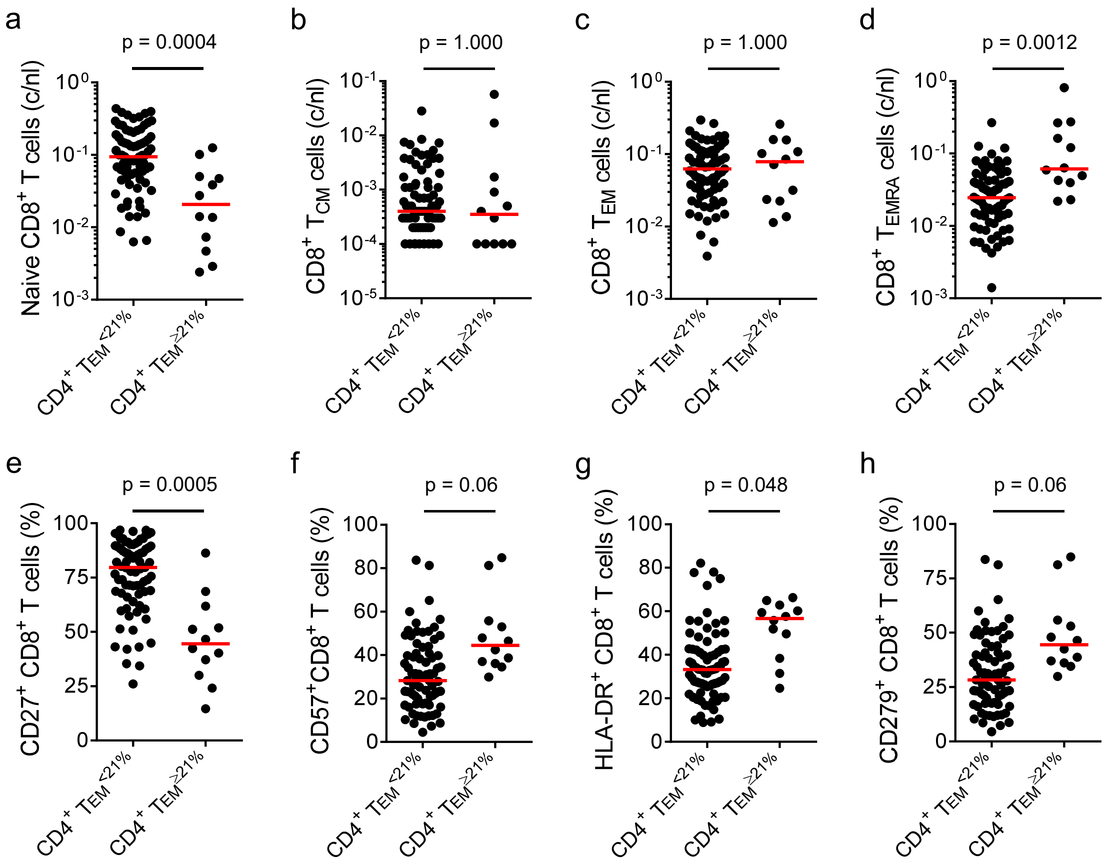

CD4+ T$_{EM}$ cell enrichment is associated with chronic activation and expansion of effector memory CD8+ T cells. **(a)** Baseline naïve CD8+ T cell counts (n=89; M.W.; Bonferroni-corrected p-value, m=4). **(b)** Baseline CD8+ T$_{CM}$ cell counts (n=89; M.W.; Bonferroni-corrected p-value, m=4). **(c)** Baseline CD8+ T$_{EM}$ cell counts (n=89; M.W.; Bonferroni-corrected p-value, m=4). **(d)** Baseline CD8+ T$_{EMRA}$ cell counts (n=89; M.W.; Bonferroni-corrected p-value, m=4). **(e)** Baseline CD27+ CD8+ T cell frequencies (n=89; M.W.; Bonferroni-corrected p-value, m=60). **(f)** Baseline CD57+ CD8+ T cell frequencies (n=89; M.W.; Bonferroni-corrected p-value, m=60). **(g)** Baseline HLA-DR+ CD8+ T cell frequencies (n=89; M.W.; Bonferroni-corrected p-value, m=60). **(h)** Baseline CD279+ CD8+ T cell frequencies (n=89; M.W.; Bonferroni-corrected p-value, m=60). Flow cytometry gating strategies are illustrated in protocols available through Protocol Exchange[1].

SUPPLEMENTARY FIGURE 10

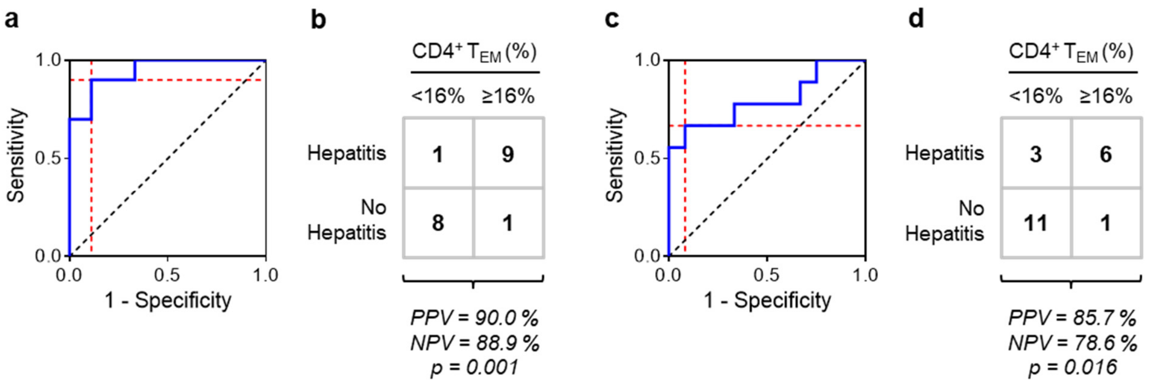

A refined prediction of hepatitis in CMV IgG$^+$ patients. **(a)** ROC analysis of CD4$^+$ T_{EM} frequency as a discriminatory marker for αPD-1/αCTLA-4-related hepatitis in CMV IgG$^+$ patients from the training set (n=19). An optimal cut-off of CD4$^+$ T_{EM} ≥16 % was established. **(b)** Classification of training set patients according to the revised cut-off of CD4$^+$ T_{EM} ≥16 % (n=19; F.E.; unadjusted p-value). **(c)** ROC analysis of CD4$^+$ T_{EM} frequency as a discriminatory marker for treatment-related hepatitis in CMV IgG$^+$ patients from the validation set (n=21). **(d)** Prediction of αPD-1/αCTLA-4-related hepatitis in training set patients according to the revised cut-off of CD4$^+$ T_{EM} ≥16 % (n=21; F.E.; unadjusted p-value).

SUPPLEMENTARY FIGURE 11

a

Laser	488 nm	561 nm				638 nm			405 nm				808 nm
Filter	525/40	585/42	610/20	710/50	763/43	660/10	712/25	763/43	450/45	525/40	610/20	763/43	885/40
Fluorochrome	AF488	PE	AF594	PC5.5	PC7	AF647	AF700	AFire750	BV421	BV510	BV605	BV785	Viability
**Antigen**	**CD45RA**	**IL-4**	**CD8**	**PD1**	**CCR7**	**IL-17A**	**CD4**	**CD3**	**IFNγ**	**TNFα**	**CD69**	**CD152**	**Live-Dead**
Clone	ALB11	MP4-2502	RPA-T8	PD1.3	G043H7	BLIG8	13B8.2	UCHT1	B27	mAb11	FN50	BNI3	na
Isotype	mIgG1	rIgG1	mIgG1	mIgG2b	mIgG2a	mIgG1	mIgG1	mIgG1	mIgG1	IgG1	mIgG1	mIgG2a	na
Fluorochrome	FITC	PE	AF594	PECy5.5	PECy7	AF647	AFA700	AFA750	BV421	AF594	BV605	BV785	ViaKr-808
Supplier	BC	BioLegend	BioLegend	BC	BioLegend	BioLegend	BC	BC	BioLegend	BioLegend	BioLegend	BioLegend	BC
Volume/test (µl)	2.4	0.4	0.23	3.0	1.50	2.0	3.0	2.4	2.0	2.0	1.5	2.0	
Extra-/intra-cellular	Extra	Intra	Extra	Extra	Extra	Intra	Extra	Extra	Intra	Intra	Extra	Intra	na
Cat.#	A07786	500810	301056	B36123	353226	512310	B10824	A94680	506538	502950	310938	369624	C36628
RRID		AB_315129	AB_2563232		AB_11126145	AB_961388			AB_2801098	AB_2565860	AB_2562307	AB_2810582	
Status	CE	RUO	RUO	ASR	RUO	RUO	ASR	CE	RUO	RUO	RUO	RUO	RUO

b

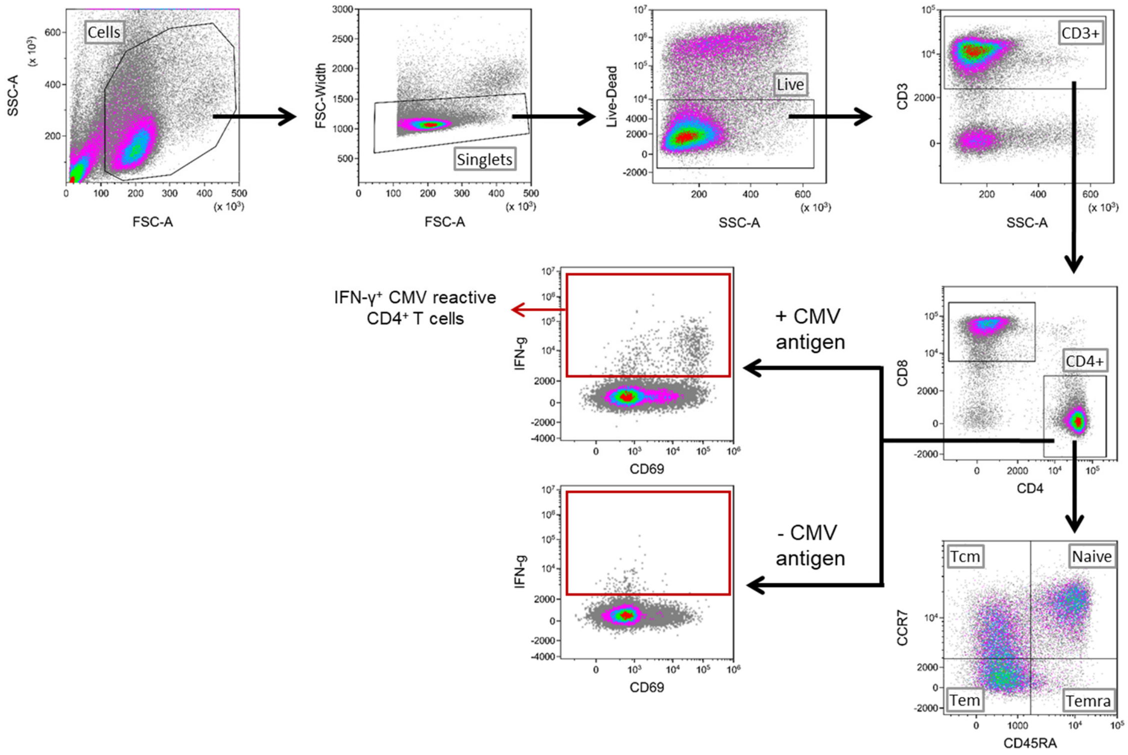

Quantifying *in vitro*-restimulated CMV-reactive T cells by flow cytometry. **(a)** 13-colour panel. A detailed step-by-step procedure for preparing and analysing samples by flow cytometry is available through Nature Protocol Exchange[2]. **(b)** This gating strategy was used to generate data presented in Figure 6 and Supplementary Figure 12.

SUPPLEMENTARY FIGURE 12

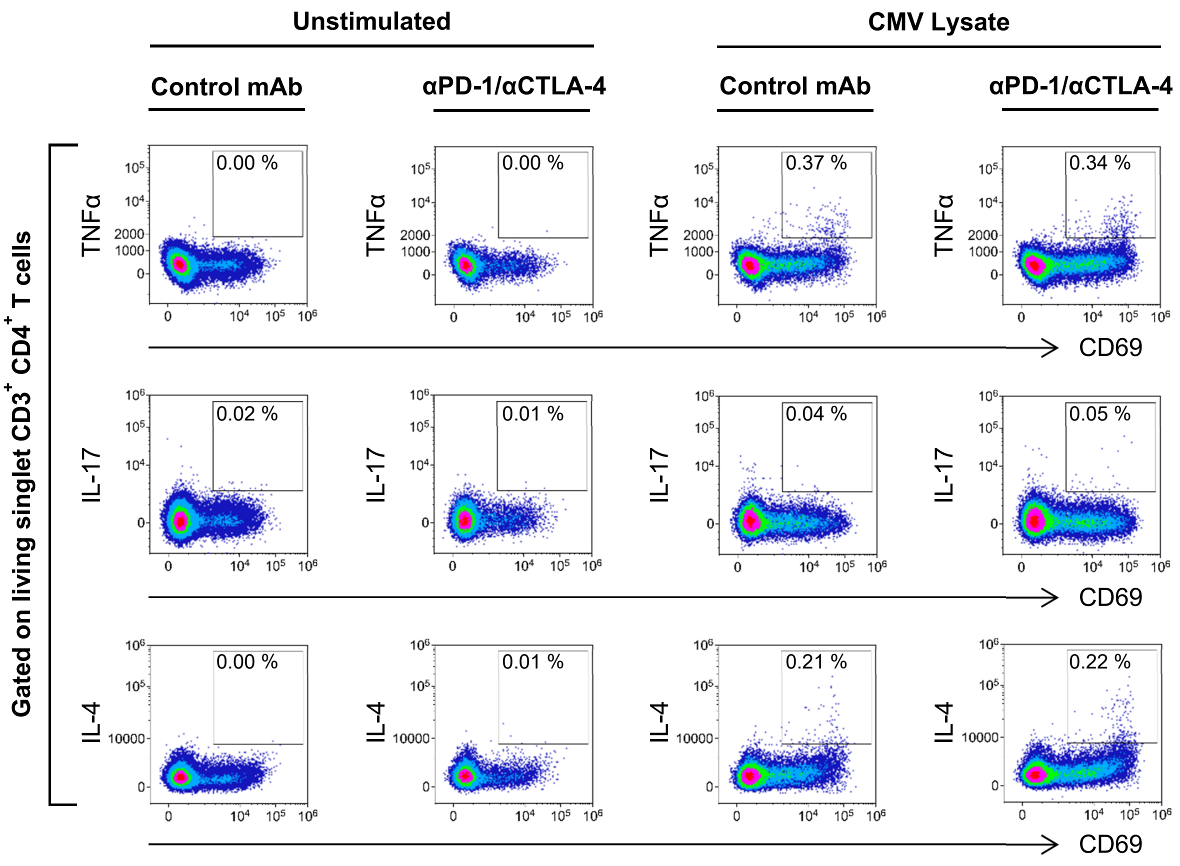

Detection of CMV-reactive TNFα- and IL-4-producing $CD4^+$ T cells in a CMV IgG^+ $CD4^+$ $T_{EM}^{\geq 16\%}$ patient who developed hepatitis. CMV-reactive $CD4^+$ T cells in patients with unresectable metastatic melanoma were assayed by in vitro stimulation with CMV lysates. Neutralising antibodies were used to detect T cells unable to respond to CMV antigens owing to expression of PD-1 or CTLA-4. Responder T cells were detected by flow cytometry analysis of TNFα, IL-17 and IL-4 expression. A detailed step-by-step procedure for preparing and analysing CMV-reactive T cells by flow cytometry is available through Nature Protocol Exchange[2].

SUPPLEMENTARY TABLE 1

	Training Set (n=44)	Validation Set (n=45)	p-value
**Patient demographics**			
Mean age (years) [95% CI]	59.0 [55.0 – 63.0]	60.4 [56.4 – 64.5]	0.560
Sex distribution	31 ♂ / 13 ♀	25 ♂ / 20 ♀	0.189
Mean BMI [95% CI]	27.3 [25.3 – 29.3]	27.6 [26.2 – 29.0]	0.617
**Baseline Biochemistry**			
Mean AST (U/l) [95% CI]	42.4 [30.9 – 54.0]	50.8 [30.8 – 70.9]	0.844
Mean ALT (U/l) [95% CI]	26.6 [18.9 – 34.2]	22.3 [19.4 – 25.2]	0.780
Mean γGT (U/l) [95% CI]	45.5 [30.3 – 60.6]	31.4 [27.1 – 35.8]	0.365
Mean total bilirubin (mg/dl) [95% CI]	0.46 [0.40 – 0.53]	0.52 [0.41 – 0.63]	0.988
CRP (mg/l) [95% CI]	20.5 [9.7 – 31.2]	29.4 [11.6 – 47.2]	0.480
LDH (U/l) [95% CI]	297 [212 – 382]	222 [195 – 249]	0.466
Protein S100 (µg/l) [95% CI]	0.65 [0.24 – 1.07]	0.26 [0.05 – 0.47]	0.329
**Baseline Haematology**			
Mean leucocytes (c/nl) [95% CI]	7.54 [6.80 – 8.28]	7.72 [6.06 – 9.38]	0.222
Mean neutrophils (c/nl) [95% CI]	5.02 [4.38 – 5.66]	5.54 [3.90 – 7.12]	0.461
Mean monocytes (c/nl) [95% CI]	0.69 [0.61 – 0.77]	0.58 [0.50 – 0.65]	**0.046**
Mean lymphocytes (c/nl) [95% CI]	1.68 [1.45 – 1.91]	1.43 [1.25 – 1.61]	0.168
Mean $CD3^+$ T cells (c/nl) [95% CI]	1.51 [1.24 – 1.79]	1.25 [1.07 – 1.42]	0.278
Mean $CD4^+$ T cells (c/nl) [95% CI]	1.02 [0.86 – 1.18]	0.87 [0.73 – 1.00]	0.214
Mean $CD8^+$ T cells (c/nl) [95% CI]	0.38 [0.28 – 0.47]	0.28 [0.22 – 0.34]	0.129
**Baseline Virology**			
HBsAg seropositive	0 (0%)	0 (0%)	1.000
HBcAg seropositive	2 (4.5%)	1 (2.2%)	0.616
HCV seropositive	0 (0%)	0 (0%)	1.000
HEV seropositive	19 of 32 tested (54.3%)	17 of 30 tested (59.4%)	1.000

Baseline Biochemistry and Haematology of patients included in the training and validation sets. Groups were compared using Fisher's Exact Test or the Mann-Whitney test. P-values were not adjusted for multiple comparison.

SUPPLEMENTARY TABLE 2

	Training Set (n=44)	Validation Set (n=45)	p-value
**Indices of tumour burden**			
Number of organs affected			
1	3 (6.8%)	8 (17.8%)	
2	19 (43.2%)	19 (42.2%)	
3	4 (9.1%)	8 (17.8%)	
4	11 (25.0%)	7 (15.6%)	0.367
5	3 (6.8%)	1 (2.2%)	
6	1 (2.3%)	1 (2.2%)	
7	2 (4.5%)	0 (0%)	
Organs with metastasis >1.5 cm			
0	19 (43.2%)	21 (46.7%)	
1	16 (36.4%)	20 (44.4%)	
2	6 (13.6%)	3 (6.7%)	0.226
3	3 (6.8%)	0 (0%)	
4	0 (0%)	1 (2.2%)	
Mean diameter of largest metastasis (cm) [95% CI]	2.91 [1.96 – 3.86]	2.31 [1.80 – 2.82]	0.640
Hepatic metastasis	15 (34.1%)	11 (24.4%)	0.358
**Treatment before study**			
Surgical excision	40 (90.9%)	42 (93.9%)	0.714
Radiotherapy	20 (45.5%)	16 (35.6%)	0.392
Checkpoint inhibitor monotherapy	5 (11.4%)	4 (8.9%)	0.739
Interferon	4 (9.1%)	4 (8.9%)	1.000
BRAFi/MEKi	9 (20.5%)	8 (17.8%)	0.793
T-VEC	2 (4.5%)	3 (6.7%)	0.511
Any drug therapy	15 (34.1%)	14 (31.1%)	0.523
**Treatment-related complications**			
Hepatitis (CTCAE grade ≥2)	18 (40.9%)	20 (44.4%)	0.831
Colitis (requiring intervention)	12 (27.3%)	17 (37.8%)	0.367
Thyroiditis	12 (27.3%)	18 (40.0%)	0.263
**Clinical response at 12 weeks**			
Progressive disease	21 (47.7%)	15 (33.3%)	
Stable disease	8 (18.2%)	3 (6.7%)	**0.050**
Partial response	11 (25.0%)	15 (33.3%)	
Complete response	4 (9.1%)	12 (26.7%)	

Clinical characteristics of patients included in the training and validation sets. Groups were compared using Fisher's Exact Test or the Mann-Whitney test. P-values were not adjusted for multiple comparison.

SUPPLEMENTARY TABLE 3

Table 3a.

	Colitis (+)				Colitis (-)				Totals
	Thyroiditis (+)		Thyroiditis (-)		Thyroiditis (+)		Thyroiditis (-)		
	Hepatitis (+)	Hepatitis (-)	Hepatitis (+)	Hepatitis (-)	Hepatitis (+)	Hepatitis (-)	Hepatitis (+)	Hepatitis (-)	
Progressive Disease	1	2	3	1	4	3	10	12	12
Stable Disease	1	0	2	3	0	1	1	3	3
Partial Response	1	3	2	2	1	5	3	9	9
Complete Response	2	3	0	3	3	0	4	1	1
Totals	5	8	7	9	8	9	18	25	89

Table 3b.

	Hepatitis	Thyroiditis	Colitis
Clinical Response	0.185	0.295	0.058
Colitis	1.000	0.153	
Thyroiditis	1.000		

No associations were found between common adverse reactions and clinical response. **(a)** Incidence of hepatitis, colitis, thyroiditis and clinical responses in a cohort of 89 patients with metastatic melanoma treated with αPD-1/αCTLA-4 dual therapy. **(b)** Pairwise p-values calculated with Fisher's exact test for associations between adverse reactions and clinical responses (n=89). No significant associations were detected. P-values were not adjusted for multiple comparison.

SUPPLEMENTARY TABLE 4

	No Hepatitis (n=51)	Hepatitis (n=38)	p-value
**Patient demographics**			
Mean age (years) [95% CI]	63.0 [59.6 – 66.5]	55.3 [50.9 – 59.6]	**0.006**
Sex distribution	35 ♂ / 16 ♀	21 ♂ / 17 ♀	0.268
Mean BMI [95% CI]	28.3 [26.5 – 30]	26.3 [24.8 – 27.9]	0.091
**Baseline Biochemistry**			
Mean AST (U/l) [95% CI]	47.5 [29.3 – 65.7]	45.8 [33.0 – 58.5]	0.324
Mean ALT (U/l) [95% CI]	23.1 [19.8 – 26.4]	26.1 [17.6 – 34.6]	0.942
Mean γGT (U/l) [95% CI]	31.1 [26.1 – 36.2]	48.1 [31.2 – 65.0]	**0.029**
Mean total bilirubin (mg/dl) [95% CI]	0.52 [0.43 – 0.60]	0.46 [0.37 – 0.54]	0.442
CRP (mg/l) [95% CI]	20.0 [10.6 – 29.5]	31.1 [10.9 – 51.3]	0.552
LDH (U/l) [95% CI]	278 [204 – 353]	235 [201 – 268]	0.885
Protein S100 (µg/l) [95% CI]	0.53 [0.17 – 0.89]	0.35 [0.09 – 0.61]	0.578
**Baseline Haematology**			
Mean leucocytes (c/nl) [95% CI]	7.98 [6.58 – 9.38]	7.17 [6.16 – 8.18]	0.382
Mean neutrophils (c/nl) [95% CI]	5.65 [4.25 – 7.05]	4.80 [3.91 – 5.68]	0.287
Mean monocytes (c/nl) [95% CI]	0.68 [0.61 – 0.75]	0.56 [0.47 – 0.65]	**0.013**
Mean lymphocytes (c/nl) [95% CI]	1.49 [1.31 – 1.67]	1.64 [1.40 – 1.88]	0.197
Mean $CD3^+$ T cells (c/nl) [95% CI]	1.36 [1.19 – 1.54]	1.40 [1.10 – 1.70]	0.846
Mean $CD4^+$ T cells (c/nl) [95% CI]	0.95 [0.83 – 1.07]	0.93 [0.74 – 1.12]	0.479
Mean $CD8^+$ T cells (c/nl) [95% CI]	0.32 [0.25 – 0.38]	0.34 [0.24 – 0.44]	0.730
**Baseline Virology**			
HBsAg seropositive	0 (0%)	0 (0%)	1.000
HBcAg seropositive	2 (3.9%)	1 (2.6%)	0.566
HCV seropositive	0 (0%)	0 (0%)	1.000
HEV seropositive	17 of 31 tested (54.8%)	21 of 36 tested (58.3%)	0.809

Baseline Biochemistry and Haematology of patients with or without hepatitis. Groups were compared using Fisher's Exact Test or the Mann-Whitney test. P-values were not adjusted for multiple comparison. The difference in γGT levels between patients who did or did not develop hepatitis is not clinically meaningful and median values in both groups lie within the normal range.

SUPPLEMENTARY TABLE 5

	No Hepatitis (n=51)	Hepatitis (n=38)	p-value
**Indices of tumour burden**			
Number of organs affected			
1	6 (11.8%)	5 (13.2%)	0.809
2	22 (43.1%)	16 (42.1%)	
3	5 (9.8%)	7 (18.4%)	
4	10 (19.6%)	8 (21.1%)	
5	3 (5.9%)	1 (2.6%)	
6	2 (3.9%)	0 (0%)	
7	1 (2.0%)	1 (2.6%)	
Organs with metastasis >1.5 cm			
0	20 (39.2%)	20 (53.6%)	0.242
1	25 (49.0%)	11 (28.9%)	
2	4 (7.8%)	5 (13.2%)	
3	1 (2.0%)	2 (5.3%)	
4	1 (2.0%)	0 (0%)	
Mean diameter of largest metastasis (cm) [95% CI]	2.40 [1.96 – 2.83]	2.89 [1.77 – 4.01]	0.622
Hepatic metastasis	15 (29.4%)	11 (31.4%)	1.000
**Treatment before study**			
Surgical excision	47 (92.2%)	35 (92.1%)	1.000
Radiotherapy	21 (41.2%)	15 (39.5%)	1.000
Checkpoint inhibitor monotherapy	4 (7.8%)	5 (13.2%)	0.488
Interferon	5 (9.8%)	3 (7.9%)	1.000
BRAFi/MEKi	10 (19.6%)	7 (18.4%)	1.000
T-VEC	1 (2.0%)	4 (10.5%)	0.159
Any drug therapy	17 (33.3%)	12 (31.6%)	1.000
**Treatment-related complications**			
Colitis (requiring intervention)	17 (33.3%)	13 (34.2%)	1.000
Thyroiditis	17 (33.3%)	12 (31.6%)	1.000
**Clinical response at 12 weeks**			
Progressive disease	18 (35.3%)	18 (47.4%)	0.185
Stable disease	7 (13.7%)	4 (10.5%)	
Partial response	19 (37.3%)	7 (18.4%)	
Complete response	7 (13.7%)	9 (23.7%)	

Baseline clinical characteristics of patients with or without hepatitis. Groups were compared using Fisher's Exact Test or the Mann-Whitney test. P-values were not adjusted for multiple comparison.

SUPPLEMENTARY TABLE 6

	No hepatitis (n=51)	Hepatitis (n=38)	Sig.
**anti-nuclear antibodies (ANA)**	31 (60.8%)	23 (60.5%)	0.576
Nuclear speckled (AC-2,-4,-5,-29)	20 (39.2%)	16 (42.1%)	0.520
AC-2	8 (15.7%)	8 (21.1%)	0.583
AC-4	11 (21.6%)	7 (18.4%)	0.109
AC-8	7 (13.7%)	0 (0%)	**0.019**
AC-18, -19, 20	3 (5.9%)	2 (5.3%)	1.000
AC-21	1 (2.0%)	0 (0%)	1.000
**anti-smooth muscle actin antibody (SMA)**	2 (3.9%)	0 (0%)	0.494
**anti-mitochondrial antibody (AMA)**	1 (2.0%)	0 (0%)	1.000
**anti-liver-kidney microsomal antibody (LKM)**	0 (0%)	0 (0%)	1.000
**anti-soluble liver antigen antibody (SLA)**	2 (3.9%)	2 (5.3%)	1.000
**anti-liver cytosol antibody (LC-1)**	0 (0%)	0 (0%)	1.000

Presence of autoimmune antibodies was not associated with development of hepatitis following αPD-1/αCTLA-4 dual therapy. Analysis of n=89 patients recruited to the training and validation sets. Fisher's Exact test. P-values were not adjusted for multiple comparison.

SUPPLEMENTARY TABLE 7

	No hepatitis (n=51)	CD4^{+} T$_{EM}^{<21\%}$ hepatitis (n=26)	CD4^{+} T$_{EM}^{\geq 21\%}$ hepatitis (n=12)	Sig.
**Patient demographics**				
Mean age (years) [95% CI]	63.0 [59.6 – 66.5]	52.0 [47.3 – 56.8]	62.3 [53.3 – 71.3]	**0.001**
Sex distribution	35 ♂ / 16 ♀	14 ♂ / 12 ♀	7 ♂ / 5 ♀	0.428
Mean BMI [95% CI]	28.3 [26.5 – 30.0]	26.5 [24.5 – 28.6]	25.8 [23.3 – 28.4]	0.237
**Baseline Biochemistry**				
Mean AST (U/l) [95% CI]	47.5 [29.3 -65.7]	38.3 [25.2 – 51.3]	63.6 [32.2 – 94.9]	0.065
Mean ALT (U/l) [95% CI]	23.1 [19.8 – 26.4]	26.5 [14.3 – 38.8]	25.2 [17.2 – 33.2]	0.677
Mean γGT (U/l) [95% CI]	31.1 [26.1 – 36.2]	46.9 [22.6 – 71.1]	50.8 [33.5 – 68.2]	**0.027**
Mean total bilirubin (mg/dl) [95% CI]	0.52 [0.43 – 0.60]	0.48 [0.36 – 0.60]	0.42 [0.33 – 0.51]	0.739
CRP (mg/l) [95% CI]	20.0 [10.6 – 29.5]	33.6 [5.2 – 62.1]	25.6 [1.5 -49.6]	0.822
LDH (U/l) [95% CI]	278 [204 – 353]	234 [190 – 278]	237 [179 – 295]	0.894
Protein S100 (ng/ml) [95% CI]	0.53 [0.17 – 0.89]	0.39 [0.02 -0.77]	0.27 [0.02 – 0.51]	0.848
**Baseline Haematology**				
Mean leucocytes (c/nl) [95% CI]	7.98 [6.58 – 9.38]	7.42 [6.06 – 8.78]	6.63 [5.14 – 8.12]	0.546
Mean neutrophils (c/nl) [95% CI]	5.65 [4.35 – 7.05]	5.03 [3.80 – 6.25]	4.30 [3.22 – 5.38]	0.510
Mean monocytes (c/nl) [95% CI]	0.68 [0.61 – 0.75]	0.55 [0.45 – 0.65]	0.60 [0.39 – 0.80]	**0.046**
Mean lymphocytes (c/nl) [95% CI]	1.49 [1.31 – 1.67]	1.67 [1.43 – 1.91]	1.58 [0.93 – 2.22]	0.258
Mean CD3^{+} T cells (c/nl) [95% CI]	1.36 [1.19 – 1.54]	1.39 [1.14 – 1.65]	1.40 [0.53 – 2.28]	0.600
Mean CD4^{+} T cells (c/nl) [95% CI]	0.95 [0.83 – 1.07]	0.96 [0.78 – 1.15]	0.85 [0.34 – 1.37]	0.290
Mean CD8^{+} T cells (c/nl) [95% CI]	0.32 [0.25 – 0.38]	0.33 [0.24 – 0.43]	0.36 [0.10 – 0.61]	0.803
**Baseline Virology**				
HBsAg seropositive	0 (0%)	0 (0%)	0 (0%)	1.000
HBcAg seropositive	1 (2.0%)	1 (3.8%)	1 (8.3%)	0.340
HCV seropositive	0 (0%)	0 (0%)	0 (0%)	1.000
HEV seropositive	19 (37.3%)	11 (42.3%)	6 (50.0%)	0.937
CMV seropositive	21 (41.2%)	8 (30.8%)	11 (91.7%)	**0.001**
**Indices of tumour burden**				
Number of organs affected				
1	6 (11.8%)	2 (7.7%)	3 (25.0%)	
2	22 (43.1%)	14 (53.8%)	2 (16.7%)	
3	5 (9.8%)	5 (19.2%)	2 (16.7%)	
4	10 (19.6%)	3 (11.5%)	5 (41.7%)	0.454
5	3 (5.9%)	1 (3.8%)	0 (0%)	
6	2 (3.9%)	0 (0%)	0 (0%)	
7	1 (2.0%)	1 (3.8%)	0 (0%)	
Organs with metastases >1.5 cm				
0	20 (39.2%)	15 (57.7%)	5 (41.7%)	
1	25 (49.0%)	7 (26.9%)	4 (33.3%)	
2	4 (7.8%)	4 (15.4%)	1 (8.3%)	0.160
3	1 (2.0%)	0 (0%)	2 (16.7%)	
4	1 (2.0%)	0 (0%)	0 (0%)	
Mean diameter of largest metastasis (cm) [95% CI]	2.40 [1.96 – 2.83]	3.24 [1.61 – 4.88]	2.11 [1.57 – 2.66]	0.846
Hepatic metastases	15 (29.4%)	8 (47.1%)	3 (25.0%)	1.000
**Treatment before enrollment**				
Surgical excision	47 (92.2%)	24 (92.3%)	11 (91.7%)	1.000
Radiotherapy	21 (41.2%)	9 (34.6%)	6 (50.0%)	0.648
Checkpoint inhibitor monotherapy	4 (7.8%)	2 (7.7%)	3 (25.0%)	0.231
Interferon	5 (9.8%)	1 (3.8%)	2 (16.7%)	0.435
BRAFi/MEKi	10 (19.6%)	3 (11.5%)	4 (33.3%)	0.295
T-VEC	1 (2.0%)	5 (19.2%)	7 (58.3%)	0.055
**Treatment-related complications**				
Colitis (requiring intervention)	34 (66.7%)	16 (61.5%)	10 (83.3%)	0.457
Thyroiditis	17 (33.3%)	8 (47.1%)	5 (41.7%)	0.816
**Clinical response at 12 weeks**				
Progressive disease	18 (35.3%)	11 (42.3%)	7 (58.3%)	
Stable disease	7 (13.7%)	3 (11.5%)	1 (8.3%)	0.143
Partial response	19 (37.3%)	7 (26.9%)	0 (0%)	
Complete response	7 (13.7%)	5 (19.2%)	4 (33.3%)	

Characteristics of patients who were classified as (i) without treatment-related hepatitis, (ii) CD4^{+} T$_{EM}^{low}$ patients who developed hepatitis, or (iii) CD4^{+} T$_{EM}^{high}$ patients who developed hepatitis. Groups were compared using Fisher's Exact Test or the Kruskall-Wallis test. P-values were not adjusted for multiple comparison.

SUPPLEMENTARY REFERENCES

1. Kronenberg, K., Riquelme, P. & Hutchinson, J. A. Standard protocols for immune profiling of peripheral blood leucocyte subsets by flow cytometry using DuraClone IM reagents. *Protocol Exchange* (2021). doi 10.21203/rs.3.pex-757/v1

2. Riquelme, P. & Hutchinson, J. A. Standard protocols for detection of human CMV-specific T cells *Protocol Exchange* (2021). doi 10.21203/rs.3.pex-758/v1
