# Supplementary material for: Virus-specific memory T cell responses unmasked by immune checkpoint blockade cause hepatitis
Source: Nat Commun. 2021 Mar 4;12:1439. doi: 10.1038/s41467-021-21572-y (PMC7933278; doi:10.1038/s41467-021-21572-y)
Supplement: Supplementary file 3 — Reporting Summary [file 41467_2021_21572_MOESM3_ESM.pdf]

nature research

Corresponding author(s): James A. Hutchinson
NCOMMS-19-36004

Last updated by author(s): Jan 27, 2021

Reporting Summary

Nature Research wishes to improve the reproducibility of the work that we publish. This form provides structure for consistency and transparency in reporting. For further information on Nature Research policies, see our Editorial Policies and the Editorial Policy Checklist.

Statistics

For all statistical analyses, confirm that the following items are present in the figure legend, table legend, main text, or Methods section.

n/a	Confirmed	
☐	☒	The exact sample size (*n*) for each experimental group/condition, given as a discrete number and unit of measurement
☐	☒	A statement on whether measurements were taken from distinct samples or whether the same sample was measured repeatedly
☐	☒	The statistical test(s) used AND whether they are one- or two-sided<br>*Only common tests should be described solely by name; describe more complex techniques in the Methods section.*
☐	☒	A description of all covariates tested
☐	☒	A description of any assumptions or corrections, such as tests of normality and adjustment for multiple comparisons
☐	☒	A full description of the statistical parameters including central tendency (e.g. means) or other basic estimates (e.g. regression coefficient) AND variation (e.g. standard deviation) or associated estimates of uncertainty (e.g. confidence intervals)
☐	☒	For null hypothesis testing, the test statistic (e.g. *F*, *t*, *r*) with confidence intervals, effect sizes, degrees of freedom and *P* value noted<br>*Give P values as exact values whenever suitable.*
☒	☐	For Bayesian analysis, information on the choice of priors and Markov chain Monte Carlo settings
☒	☐	For hierarchical and complex designs, identification of the appropriate level for tests and full reporting of outcomes
☐	☒	Estimates of effect sizes (e.g. Cohen's *d*, Pearson's *r*), indicating how they were calculated

Our web collection on statistics for biologists contains articles on many of the points above.

Software and code

Policy information about availability of computer code

Data collection	No custom software was used for data collection
Data analysis	No custom software was used for data analysis

For manuscripts utilizing custom algorithms or software that are central to the research but not yet described in published literature, software must be made available to editors and reviewers. We strongly encourage code deposition in a community repository (e.g. GitHub). See the Nature Research guidelines for submitting code & software for further information.

Data

Policy information about availability of data

All manuscripts must include a data availability statement. This statement should provide the following information, where applicable:

- Accession codes, unique identifiers, or web links for publicly available datasets
- A list of figures that have associated raw data
- A description of any restrictions on data availability

Data supporting the findings of this study are are available within the article and its supplementary information files or are available from the corresponding author subject to legal restrictions governing use of personal information.

Field-specific reporting

Please select the one below that is the best fit for your research. If you are not sure, read the appropriate sections before making your selection.

☒ Life sciences ☐ Behavioural & social sciences ☐ Ecological, evolutionary & environmental sciences

For a reference copy of the document with all sections, see nature.com/documents/nr-reporting-summary-flat.pdf

Life sciences study design

All studies must disclose on these points even when the disclosure is negative.

Sample size	In our original design, we planned to sequentially recruit n=40 informative patients to a training set to ensure 10 or more cases per outcome (ie. hepatitis versus no hepatitis) given an estimated incidence of hepatitis of 50 %. It was planned to sequentially recruit the same number of informative patients to a validation set. Following an editorial request from Nature Communications, our study was extended to n=103 patients in total. Of these, 14 patients were excluded from subsequent analyses because they significantly deviated from the treatment protocol or died before registering an end-point of complication-free survival to staging at 12 weeks or were treated with prophylactic valganciclovir. Hence, n=89 informative patients were ultimately included in the training and validation cohorts. To minimise selection biases, these cases were randomised to new training and validation sets. The validation set confirmed CD4+ Tem frequency in CMV-infected individuals as a statistically significant discriminator of patients at risk of hepatitis (AUROC=0.885; PPV=88.2%; NPV=82.6%; Fisher's Exact test, p=1.4x10E-5) and demonstrated the adequate size of our samples. In Figure 9 we present 4 patients treated with prophylactic valganciclovir who were not included in the training or validation sets. In Figure 2e, we compare all patients with metastatic melanoma receiving dual therapy to n=49 patients with fully resectable melanoma receiving monotherapy. Finally, we also consider n=4 patients with unresectable melanoma who were electively treated with anti-PD1 monotherapy (Figure 2j). In total, we report data from n=160 patients.
Data exclusions	107 adults (>18 years) presenting with unresectable metastatic melanoma who were suitable for treatment with anti-PD-1/anti-CTLA-4 dual therapy were screened for trial eligibility. 103 patients were recruited in total. Of these, 14 patients were excluded from subsequent analyses because they significantly deviated from the treatment protocol or died before registering an end-point of complication-free survival to staging at 12 weeks or were treated with prophylactic valganciclovir (please refer to Supplementary Materials 1).
Replication	We report a training set (n=44) and independent validation set (n=45) to demonstrate replicability. As shown in Figures 2b and 2c, the key finding from the training set that CD4+ effector memory T cell frequency distinguishes a subset of patients predisposed to checkpoint blockade-related hepatitis was confirmed in the validation set.
Randomization	89 eligible, informative patients were randomised equally to the training and validation sets.
Blinding	(I) Analysis of clinical flow cytometry data (including estimation of CD4+ Tem %) was performed by an experienced operator (coauthor K.K). who was blinded to clinical information about the patient. (II) During the model building and verification phase of this study, the clinical investigators were blinded to immune phenotyping data (including CD4+ Tem status) about patients. (III) When patients received Valganciclovir treatment or prophylaxis, the responsible clinical investigators knew the patients' CD4+ Tem status and CMV IgG serology results, and were not blinded to treatment with Valganciclovir. (IV) The scientist (coauthor P.R.) who conducted the CMV-reactive T cell assays shown in Figure 6 was blinded to the CMV status and clinical outcome of the patients during the experiment and extraction of results from flow cytometry data.

Reporting for specific materials, systems and methods

We require information from authors about some types of materials, experimental systems and methods used in many studies. Here, indicate whether each material, system or method listed is relevant to your study. If you are not sure if a list item applies to your research, read the appropriate section before selecting a response.

Materials & experimental systems

n/a	Involved in the study	
☐	☒	Antibodies
☒	☐	Eukaryotic cell lines
☒	☐	Palaeontology and archaeology
☒	☐	Animals and other organisms
☐	☒	Human research participants
☐	☒	Clinical data
☒	☐	Dual use research of concern

Methods

n/a	Involved in the study	
☒	☐	ChIP-seq
☐	☒	Flow cytometry
☒	☐	MRI-based neuroimaging

Antibodies

Antibodies used	(I) Antibodies used for marker detection in flow cytometry are described below in the 'Flow Cytometry' section, as well as the detailed step-by-step protocols provided as appendices to this Reporting Summary. (II) Neutralising monoclonal antibodies against

	PD-1 (MAB10864, R&D Systems) and CTLA-4 (MAB3254, R&D) or an equivalent concentration of isotype control antibody (MAB002; R&D) were added to CMV-stimulated T cell assays in some conditions.
Validation	(I) Please refer to our separate technical publications in Protocol Exchange, as well as Streitz-M, Transpl. Res. (2013) and Kverneland-AH, Cytometry A (2016). (II) The manufacturer provides batch-specific performance data for these functional antibodies.

Human research participants

Policy information about studies involving human research participants

Population characteristics	Study samples were obtained from adult patients with Stage III or IV melanoma receiving standard-of-care treatment with anti-PD-1 monotherapy or combined therapy according to local guidelines. Stage IV patients with unresectable metastatic disease who received first- or second-line checkpoint inhibitor therapy were initially treated with Nivolumab (anti-PD-1; Bristol-Myers Squibb) and Ipilimumab (anti-CTLA-4; Bristol-Myers Squibb) for four cycles, and thereafter with Nivolumab maintenance therapy (3 mg/kg at 3 week intervals). Those patients with complete resection of Stage III melanoma who received adjuvant checkpoint inhibitor therapy were treated for up to 1 year with Pembrolizumab (anti-PD-1; MSD) or Nivolumab. Patient characteristics (age, sex, staging, previous therapies, etc.) are summarised in Supplementary Materials 2, 3, 4, 5, 6, 7 & 8.
Recruitment	All adult patients with Stage III or IV melanoma considered for treatment with anti-PD-1 monotherapy or combined anti-PD-1 plus anti-CTLA-4 therapy who presented to the Department of Dermatology outpatient clinic were invited to participate in our study. 107 sequentially presenting patients were screened and only 4 were ineligible or non-consenting. 89 patients were recruited. Because study uptake was so high, we do not consider self-selection as a relevant confounder. We are not aware of any selection biases that might have impacted our results.
Ethics oversight	This study was approved by the Ethics Commission of the University of Regensburg (https://www.uni-regensburg.de/ethikkommission) through Ethics Vote 16-101-0125. The Regensburg Ethics Commission can be contacted by writing to Universität Regensburg, Ethikkommission, 93040 Regensburg or sending an email to ethikkommission@ur.de or by calling +49-941-943-5370.

Note that full information on the approval of the study protocol must also be provided in the manuscript.

Clinical data

Policy information about clinical studies

All manuscripts should comply with the ICMJE guidelines for publication of clinical research and a completed CONSORT checklist must be included with all submissions.

Clinical trial registration	This study was registered with clinicaltrials.gov (NCT04158544).
Study protocol	All relevant documentation is available from the Principal Investigator upon request.
Data collection	Specimens were obtained from patients with Stage III or IV melanoma participating in a single-center observational clinical trial. The first reported patient was recruited in October 2016 and the last reported patient was recruited in July 2020. Pre-treatment blood samples were collected in the Interdisciplinary Center for Tumour Therapy (ICT) at University Hospital Regensburg (UKR). Routine clinical analyses were performed by the Institute of Clinical Chemistry and Laboratory Medicine or Institute of Clinical Microbiology and Hygiene at UKR. Flow cytometry samples were processed by the Department of Surgery in a GMP-compliant analytical laboratory environment. Clinical data were collated from hospital records by the responsible clinical investigators.
Outcomes	The purpose of this study was to collect clinical and research data about complications occuring after anti-PD-1/CTLA-4 therapy in patients with advanced melanoma. The primary outcome of this study was incidence of hepatitis within 1 year of first dose. Other clinical response variables included: time-to-hepatitis, severity of hepatitis (CTCAE score), incidence of colitis, severity of colitis, incidence of thyroiditis, death and clinical response. Clinical investigations at baseline included routine Biochemistry and Haemtology, virological investigations for HBV, HCV, HEV, CMV, EBV, HSV, TTV and adenovirus, profiling of autoimmune antibodies and routine imaging for tumour staging. As potential explanatory variables for predisposition for hepatitis, data from immune profiling by flow cytometry were collected.

Flow Cytometry

Plots

Confirm that:

- [x] The axis labels state the marker and fluorochrome used (e.g. CD4-FITC).
- [x] The axis scales are clearly visible. Include numbers along axes only for bottom left plot of group (a 'group' is an analysis of identical markers).
- [x] All plots are contour plots with outliers or pseudocolor plots.
- [x] A numerical value for number of cells or percentage (with statistics) is provided.

Methodology

Sample preparation	Detailed standard operating procedures for processing, measuring and analysing clinical blood samples are provided separately through Protocol Exchange. Copies of these protocols are provided as appendices to this Reporting Summary. In

	brief, peripheral blood samples were collected into EDTA-vacutainers by venepuncture and then delivered to the immune monitoring laboratory at ambient temperature. Pre-analytical samples were stored for up to 4 hours at 4°C until processing. Whole blood was stained with DuraClone reagents (Duraclone IM Phenotyping Basic Tube, B53309; Duraclone IM T cell Subsets Tube, B53328; Duraclone IM TCRs Tube, B53340; Duraclone IM Treg Tube, B53346; Duraclone IM B cells Tube, B53318; Duraclone IM Dendritic Cells Tube, B53351; all from Beckman Coulter, Krefeld, Germany).
Instrument	Navios (Serial Nr. AT26208) or Navios EX (Serial Nr. BA14016) from Beckman Coulter or CytoFlex LX
Software	Data were recorded with Cytometry List Mode Data Acquisition and Analysis Software version 1.3 (Beckman Coulter) or CytExpert. Blinded analyses were performed by an experienced operator using Kaluza version 2.1.
Cell population abundance	We surveyed 160 human leucocyte subpopulations in peripheral blood samples, including rare populations such as pDC. We also measured CMV-reactive CD4+ Tem after in vitro stimulation, which represented as few as 0.01 - 0.1 % of events.
Gating strategy	Detailed standard operating procedures for processing, measuring and analysing clinical blood samples are provided separately through Protocol Exchange. Gating strategies for all flow cytometry anaylses are provided as Supplementary Figures 2, 3, 4, 5, 6, 7, 8 & 11.

☒ Tick this box to confirm that a figure exemplifying the gating strategy is provided in the Supplementary Information.
